# Impact of the COVID-19 pandemic on sexual and reproductive health among women with induced abortion

**DOI:** 10.1038/s41598-021-95868-w

**Published:** 2021-08-11

**Authors:** Pengcheng Tu, Jianan Li, Xiaomei Jiang, Kaiyan Pei, Yiqun Gu

**Affiliations:** 1 National Research Institute for Family Planning, Beijing, China; 2 Graduate School, Chinese Academy of Medical Sciences and Peking Union Medical College, Beijing, China

**Keywords:** Health care, Medical research, Diseases

## Abstract

The coronavirus disease (COVID-19) has already been declared a global pandemic. To our knowledge, there is very little information regarding the effects of COVID-19 on women seeking reproductive health services, specifically abortion. This study was aimed to assess the impact of the COVID-19 pandemic on reproductive and sexual health among women seeking abortion services. We conducted a series of preliminary analyses using data collected from ten maternal and child health hospitals of seven provinces in China before and during the COVID-19 lockdown. The present study showed that a significant decrease was observed in the frequency of sexual intercourse during the COVID-19 pandemic. Moreover, a significant increase in contraceptive use including condom, rhythm method and coitus interruptus whereas a decrease in choosing oral contraceptives were observed during the COVID-19 pandemic. In addition, the pandemic was associated with increased intention of seeking induced abortion due to social factors. Future research should look into the long-term impact of the COVID-19 pandemic on sexual and reproductive health.

## Introduction

As an event that can cause physical, emotional, and psychological harm, the COVID-19 pandemic may affect sexual and reproductive health of individuals. From the end of January 2020, people in China were required to implement “social distancing” and restricted to step outside homes except for essential activities, and public transport was suspended^1,2^. Even China had a sound drug supply system, contraceptives in some areas were out of stock or in short supply during the pandemic^3^.

In addition, many provinces have implemented guidelines to reduce pressure on the health care system, including the suspension of nonemergency medical care and elective surgeries. Thus many hospitals have reduced or stopped provision of reproductive health services during the COVID-19 pandemic. These restrictive social measures that are important for the COVID-19 control but restrictions in movement, social isolation and lack of availability of reproductive health services could lead to reduced access to abortion services and could have a profound influence on sexual and reproductive health^4–6^. Studies have showed that many women are unable to obtain family planning services in order to avoid unwanted pregnancies during some other public health emergencies^7,8^. Meanwhile, women’s sexual activity and contraceptive use may have to adapt under these unprecedented conditions. They may lose support and comfort during the pandemic because of social isolation from friends and relatives^9^. To our knowledge, there is very little information regarding the effects of COVID-19 on women seeking reproductive health services, specifically abortion.

This study was aimed to assess the impact of the COVID-19 pandemic on reproductive and sexual health among women seeking abortion services. We conducted a series of preliminary analyses using data collected from ten maternal and child health hospitals of seven provinces in China before and during the COVID-19 lockdown.

## Methods

This study was based on our prospective multicenter cohort study to evaluate effects of prior induced termination of pregnancy on complications and pregnancy outcomes (ClinicalTrials.gov Identifier: NCT04183829). The cohort study is ongoing but as yet unpublished. Women who experienced induced abortion in early pregnancy (< 12 weeks of gestation) were included in this study from May 2019 in ten maternal and child health hospitals of seven provinces in China. No other specific inclusion or exclusion criteria were used. For the present study, data from the cohort were extracted. For each participant, a detailed medical history was obtained, including age, nationality, marital status, education, and household income. Other details recorded were frequency of sexual intercourse, contraceptive use in sexual encounters, reasons for abortion and the choice of abortion methods. All data were recorded prospectively in the electronic data system.

To assess the impact of the COVID-19 pandemic on women seeking abortion services and their reproductive and sexual health, we conducted an observational study using previously collected data from our cohort study described above to compare the demographic characteristics, frequency of sexual intercourse, contraceptive use in sexual encounters, reasons for abortion and the choice of abortion methods before (May 1, 2019 through January 22, 2020) and during the COVID-19 lockdown (January 23, 2020 through June 30, 2020).

All participants provided written informed consent first and for participants under the age of 18 years, informed consent was obtained from a parent and/or legal guardian. This study was approved by the Ethics Review Committee of National Research Institute for Family Planning. All methods were performed in accordance with the relevant guidelines and regulations.

### Statistical analysis

Descriptive statistics were performed on the variable of interest. We used χ^2^ tests to compare demographic and clinical characteristics of participants before and during the COVID-19 lockdown. We also used multivariable logistic regression to estimate odds ratios (ORs) and 95% CIs for the association between the COVID-19 pandemic and frequency of sexual intercourse, controlling for demographic characteristics; the model controlled for age, marital status, education and household income. Two-sided *P* < 0.05 was used to test statistical significance. We excluded missing data from all analyses, but denominators were provided to put these missing data into context. All analyses were performed using SPSS Statistics version 23 (IBM).

## Results

### Demographics

A total of 3789 participants were included in the analysis. Of these, 2054 participants (2004/2054[97.6%] the Han nationality) were included in the pre-COVID-19 sample (May 1, 2019 through January 22, 2020) and 1735 participants (1695/1735[97.7%] the Han nationality) were included in the sample during the COVID-19 lockdown (January 23, 2020 through June 30, 2020). Table 1 presents the demographic characteristics of samples before and during COVID-19 lockdown. The proportion of the participants aged 35–49 years during the COVID-19 lockdown was higher than that before the COVID-19 lockdown (370/1696[21.8%] vs 338/1996[16.9%]; *P* < 0.001). Similarly, before COVID-19, 1371/2051 (66.8%) were married, but this rised to 1325/1729 (76.6%) during lockdown (*P* < 0.001). Education levels were higher among the participants during the COVID-19 lockdown compared with the pre-COVID-19 sample (≥ College: 679/1725[39.4%] vs 664/2044[32.5%]; *P* < 0.001). But the sample during the COVID-19 lockdown had a lower income than the pre-COVID-19 sample (Household income, CNY < 5000: 990/1708[58.0%] vs 928/2043[45.4%]; *P* < 0.001).

**Table 1:** Demographic characteristics of samples before and during COVID-19 lockdown.

Variable	Before COVID-19^a^ n/N (%)	During COVID-19^b^ n/N (%)	Statistics	P value^c^
**Age, years**				
15–24	163/1996 (8.2)	169/1696 (10.0)	$\upchi _{3}^{2}$ = 23.950	< .001
25–29	726/1996 (36.4)	519/1696 (30.6)		
30–34	769/1996 (38.5)	638/1696 (37.6)		
35–49	338/1996 (16.9)	370/1696 (21.8)		
**Nationality**				
Han	2004/2054 (97.6)	1695/1735 (97.7)	$\upchi _{4}^{2}$ = 12.239	.016
Manchu	9/2054 (0.4)	3/1735 (0.2)		
Hui	5/2054 (0.2)	2/1735 (0.1)		
Mongolian	13/2054 (0.6)	25/1735 (1.4)		
Other	23/2054 (1.1)	10/1735 (0.6)		
**Marital status**				
Unmarried	645/2051 (31.4)	383/1729 (22.2)	$\upchi _{2}^{2}$ = 43.948	< .001
Married	1371/2051 (66.8)	1325/1729 (76.6)		
Widowed or divorced	35/2051 (1.7)	21/1729 (1.2)		
**Education**				
< High school	248/2044 (12.1)	183/1725 (10.6)	$\upchi _{3}^{2}$ = 22.871	< .001
High school	452/2044 (22.1)	309/1725 (17.9)		
Some college	680/2044 (33.3)	554/1725 (32.1)		
≥ College	664/2044 (32.5)	679/1725 (39.4)		
**Household income, CNY**				
< 5000	928/2043 (45.4)	990/1708 (58.0)	$\upchi _{3}^{2}$ = 60.741	< .001
5000–9999	774/2043 (37.9)	521/1708 (30.5)		
10,000–15,000	206/2043 (10.1)	123/1708 (7.2)		
> 15,000	135/2043 (6.6)	74/1708 (4.3)		

^a^n = number who answered the question; denominator is not always 2054 because of missing data.

^b^n = number who answered the question; denominator is not always 1735 because of missing data.

^c^Two-tailed χ^2^ analysis conducted for significance testing.

### Sexual activity and choice of contraceptive methods before and during COVID-19 lockdown

Sexual activity and choice of contraceptive methods before and during COVID-19 lockdown are presented in Table 2. Women reported a significantly lower frequency of sexual activity during lockdown, compared with the pre-COVID-19 sample (Sex frequency, < 1 per week: (1213/1723[70.4%] vs 884/2052[43.1%]; *P* < 0.001). Multivariable logistic regression showed that after controlling for age, marital status, education and household income, participants during COVID-19 lockdown had 2.878-fold increased odds of low sex frequency (Sex frequency, < 1 per week) compared with participants before COVID-19 lockdown (OR 2.878 [95% CI 2.485–3.332]) (Table 3). The proportion of women choosing contraception during COVID-19 lockdown was significantly higher than that of the pre-COVID-19 sample (1109/1696[65.4%] vs 875/1992[43.9%]; *P* < 0.001). Of these women choosing contraception, the proportion of choosing condom, rhythm method and coitus interruptus during COVID-19 lockdown are significantly higher when comparing with those of the pre-COVID-19 sample (Condom: 831/1109[74.9%] vs 403/875[46.1%], *P* < 0.001; rhythm method: 624/1109[56.3%] vs 309/875[35.3%], *P* < 0.001; coitus interruptus: 544/1109[49.1%] vs 175/875[20.0%], *P* < 0.001). Conversely, fewer women chose oral contraceptives during COVID-19 lockdown than the pre-COVID-19 sample (88/1109[7.9%] vs 155/875 [17.7%]; *P* < 0.001).

**Table 2:** Sexual activity and choice of contraceptive methods before and during COVID-19 lockdown.

Variable	Before COVID-19^a^ n/N (%)	During COVID-19^b^ n/N (%)	Statistics	P value^c^
**Sex frequency, per week**				
< 1	884/2052 (43.1)	1213/1723 (70.4)	$\upchi _{2}^{2}$ = 286.125	< .001
1–2	1013/2052 (49.4)	459/1723 (26.6)		
≥ 3	155/2052 (7.6)	51/1723 (3.0)		
**Contraception**				
Yes	875/1992 (43.9)	1109/1696 (65.4)	$\upchi _{1}^{2}$ = 169.783	< .001
No	1117/1992 (56.1)	587/1696 (34.6)		
**Condom**				
Yes	403/875 (46.1)	831/1109 (74.9)	$\upchi _{1}^{2}$ = 173.443	< .001
No	472/875 (53.9)	278/1109 (25.1)		
**Rhythm method**				
Yes	309/875 (35.3)	624/1109 (56.3)	$\upchi _{1}^{2}$ = 86.193	< .001
No	566/875 (64.7)	485/1109 (43.7)		
**Coitus interruptus**				
Yes	175/875 (20.0)	544/1109 (49.1)	$\upchi _{1}^{2}$ = 178.669	< .001
No	700/875 (80.0)	565/1109 (50.9)		
**Oral contraceptive**				
Yes	155/875 (17.7)	88/1109 (7.9)	$\upchi _{1}^{2}$ = 43.519	< .001
No	720/875 (82.3)	1021/1109 (92.1)		

^a^n = number who answered the question; denominator is not always 2054 because of missing data.

^b^n = number who answered the question; denominator is not always 1735 because of missing data.

^c^Two-tailed χ^2^ analysis conducted for significance testing.

**Table 3:** Correlates of sexual frequency among subjects as determined logistic regression analysis.

Variables	Univariate analysis	Multivariate analysis^a^	
	P value	OR (95%CI)	P value
**Age, years**			
15–24	< .001	1 [Reference]	
25–29		0.620 (0.471–0.818)	.001
30–34		0.771 (0.576–1.031)	.079
35–49		1.035 (0.751–1.427)	.833
**Marital status**			
Unmarried	< .001	1 [Reference]	
Married		1.694 (1.415–2.029)	< .001
Widowed or divorced		1.124 (0.617–2.049)	.702
**Education**			
< High school	.021	1 [Reference]	
High school		0.975 (0.750–1.268)	.850
Some college		1.255 (0.980–1.607)	.071
≥ College		1.643 (1.280–2.108)	< .001
**Household income, CNY**			
< 5000	< .001	1 [Reference]	
5000–9999		0.495 (0.421–0.581)	< .001
10,000–15,000		0.143 (0.107–0.190)	< .001
> 15,000		0.203 (0.146–0.282)	< .001
**Period**			
Before COVID-19	< .001	1 [Reference]	
During COVID-19		2.878 (2.485–3.332)	< .001

^a^Complete case analysis used for multiple logistic regression resulting included 3624 participants for this model.

### Reasons for abortion and the choice of abortion methods

Table 4 presents the reasons for abortion and the choice of abortion methods before and during COVID-19 lockdown. The proportion of women seeking abortion services due to social factors during COVID-19 lockdown was significantly higher than that of the pre-COVID-19 sample (1349/1700[79.4%] vs 1275/1999[63.8%]; *P* < 0.001). No statistical differences were observed in choice of abortion methods before and during COVID-19 lockdown (*P* = 0.360). Most women chose surgical abortion (Before COVID-19: 1742/2003[87.0%] vs during COVID-19: 1492/1696[88.0%]) rather than medical abortion (Before COVID-19: 261/2003[13.0%] vs during COVID-19: 204/1696 [12.0%]).

**Table 4:** Reasons for abortion and the choice of abortion methods before and during COVID-19 lockdown.

Variable	Before COVID-19^a^ n/N (%)	During COVID-19^b^ n/N (%)	Statistics	P value^c^
**Reasons for abortion**				
Social factors	1275/1999 (63.8)	1349/1700 (79.4)	$\upchi _{2}^{2}$ = 109.359	< .001
Medical concerns	708/1999 (35.4)	347/1700 (20.4)		
Policy issues	16/1999 (0.8)	4/1700 (0.2)		
**Abortion methods**				
Surgical abortion	1742/2003 (87.0)	1492/1696 (88.0)	$\upchi _{1}^{2}$ = 0.839	.360
Medical abortion	261/2003 (13.0)	204/1696 (12.0)		

^a^n = number who answered the question; denominator is not always 2054 because of missing data.

^b^n = number who answered the question; denominator is not always 1735 because of missing data.

^c^Two-tailed χ^2^ analysis conducted for significance testing.

## Discussion

This study provided preliminary evidence of the impact of the COVID-19 pandemic on the sexual and reproductive health of women with induced abortion. According to the data obtained from the study, as the proportion of participants aged over 35 years, married, with a college degree or above increased during the COVID-19 pandemic, a decrease in the frequency of sexual intercourse was observed. Moreover, the participants during COVID-19 lockdown had 2.878-fold increased odds of low sex frequency (Sex frequency, < 1 per week) compared with participants before COVID-19 lockdown (OR 2.878 [95% CI 2.485–3.332]). These findings partly corresponded to previous studies^10,11^. An online survey in China showed that 44% of participants reported a decrease in the number of sexual partners and about 37% of participants reported a decrease in sexual frequency during the COVID-19 pandemic^10^. Fuchs et al. also found that during the pandemic, the frequency of sexual intercourse declined compared to the period before^11^. There are two main reasons which may account for these findings. First of all, quarantine made it harder to reach the preferred sexual partner during the pandemic. Furthermore, women’s stress about a possible pregnancy due to the fear of economic instability and lack of knowledge of pregnancy outcomes related to the COVID-19 infection might lead to the decreased desire for sexual intercourse. However, a recent study conducted in Italy reported no reduction in frequency of sexual intercourse during the COVID-19 pandemic, with no significant differences among genders^12^. In comparison, another study conducted in Turkey found that the COVID-19 pandemic is associated with increased sexual desire and frequency of intercourse, whereas quality of sexual life significantly decreased^13^.

Our study also demonstrated that there was a significant increase in the rate of participants using contraceptive methods during the pandemic (1109/1696[65.4%] vs 875/1992[43.9%]; *P* < 0.001). Of these, more women chose condom, rhythm method and coitus interruptus during COVID-19 lockdown (Condom: 831/1109[74.9%] vs 403/875[46.1%], *P* < 0.001; rhythm method: 624/1109[56.3%] vs 309/875[35.3%], *P* < 0.001; coitus interruptus: 544/1109[49.1%] vs 175/875[20.0%], *P* < 0.001) whereas fewer chose oral contraceptives (88/1109[7.9%] vs 155/875 [17.7%]; *P* < 0.001). Limited public transport and fear of contracting the virus during the lockdown may result in delays in seeking oral contraceptive in a pharmacy. Meanwhile, the distribution of oral contraceptives to retail sellers may be impacted by the suspended transportation between cities during the lockdown. Li et al. found that even in a country with a sound drug supply system such as China, there were still a shortage of contraceptives in some areas during the pandemic^3^.

In addition, we found that the proportion of women seeking abortion due to social factors increased significantly during the pandemic (1349/1700[79.4%] vs 1275/1999[63.8%];* P* < 0.001). Childbirth, as one of the most important events in a person’s life, is naturally associated with a certain level of worry. Yet, as evidenced by our results, abortion due to social factors soared during the pandemic, indicating that the pandemic has the potential to increase additional stress in the pregnant population. This finding is in agreement with two recent studies. A large-scale longitudinal survey conducted in Sweden showed that the worries of pregnant women about their own health, as well as their child’s health remained at higher than usual levels during the pandemic^14^. A cross-sectional study conducted in Iran also revealed that because of social stress and anxiety, pregnant women want to terminate their pregnancies prematurely^15^.

We are aware that the COVID-19 pandemic is a period when countless factors might have an impact on many different aspects of human health, including sexual and reproductive health. Much attention of health workers is focused on infectious diseases and symptoms during the pandemic. From the results of this study, it is suggested that more attention should be paid to sexual and reproductive health in such an unusual situation and targeted interventions are needed to improve sexual health in addition to the physical and mental health of individuals. Additionally, the stakeholders need to take actions to guarantee a convenient access to basic reproductive health services for pepole in childbearing age during the pandemic. Moreover, telemedicine can play a significant role to facilitate contact between patients and physicians in case future pandemics or similar events may happen.

### Limitations

This study has some limitations. First, we compare the pre-COVID-19 sample and the sample during the COVID-19 lockdown rather than the same individuals over time so the pre-COVID-19 sample can provide only a proxy for the true baseline level for the sample during the COVID-19 lockdown. Further limitations include recall bias and missing data for several variables as not everyone completed all questions. While our study reaches a broad sample, it is concentrated in city areas in China and the COVID-19 pandemic is peaking in different cities at different times. This may represent a relative bias of selection. This study was not designed to assess regional differences. Finally, the study has not determined the long-term impact of the COVID-19 pandemic on sexual and reproductive health; this must be realised in subsequent cohort analyses of future waves of data.

## Conclusions

To our knowledge, this may be the first study among women with induced abortion on sexual health during the COVID-19 pandemic. The present study showed that a significant decrease was observed in the frequency of sexual intercourse during the COVID-19 pandemic. Moreover, a significant increase in contraceptive use including condom, rhythm method and coitus interruptus whereas a decrease in choosing oral contraceptives were observed during the COVID-19 pandemic. In addition, the pandemic was associated with increased intention of seeking induced abortion due to social factors. These findings demonstrate that the pandemic dramatically affects sexual health among women. Basic reproductive health services and contraceptives supply chain operations should be guaranteed to protect the rights and health of women during the COVID-19 pandemic. Future research should look into the long-term impact of the COVID-19 pandemic on sexual and reproductive health.
